# Successful treatment of out-of-hospital cardiopulmonary arrest due to streptococcal toxic shock syndrome – effectiveness of extracorporeal membrane oxygenation and the rapid antigen group A streptococcus test: a case report

**DOI:** 10.1186/s13256-018-1780-2

**Published:** 2018-09-01

**Authors:** Yukio Mizuguchi, Norimasa Taniguchi, Akihiko Takahashi

**Affiliations:** Cardiovascular Section, Sakurakai Takahashi Hospital, 5-18-1 Oikecho, Suma-ku, Kobe, Hyogo 654-0026 Japan

**Keywords:** Streptococcal toxic shock syndrome, Cardiopulmonary arrest, Extracorporeal membrane oxygenation, Rapid antigen group A streptococcus test

## Abstract

**Background:**

Streptococcal toxic shock syndrome caused by *Streptococcus pyogenes*, a group A streptococcus, infection is a rare condition that rapidly progresses to multiple organ failure, shock, and death. It is thus important to promptly establish a diagnosis, provide hemodynamic support, and initiate appropriate antibiotics therapy.

**Case presentation:**

A 70-year-old Asian man presented with ventricular fibrillation. Extracorporeal membrane oxygenation was initiated 20 minutes after admission after unsuccessful conventional cardiopulmonary resuscitation including five attempts of electrical cardioversion. On the sixth attempt, a sinus rhythm was obtained. A physical examination revealed a large abscess in his right gluteal region, and computed tomography showed a large low-density area in the right gluteus maximus. Blood examination revealed elevated levels of inflammatory markers, hepatic enzymes, creatinine, and creatinine kinase. Transthoracic echocardiography demonstrated diffuse hypokinesis with an ejection fraction of 25%. A subsequent coronary angiography revealed normal findings. Therefore, we diagnosed our patient as having septic shock and conducted surgical drainage. A rapid antigen group A streptococcus test yielded positive results, which necessitated treatment comprising benzylpenicillin and clindamycin. He was successfully weaned from extracorporeal membrane oxygenation and continuous hemodiafiltration 4 days later and ventilation 9 days later; he was later transferred to another hospital to receive a skin graft.

**Conclusions:**

Our case report is the first to demonstrate the successful treatment of cardiac arrest caused by streptococcal toxic shock syndrome via extracorporeal membrane oxygenation and prompt initiation of antibiotic therapy. The rapid antigen group A streptococcus test may be an effective approach to promptly diagnose streptococcal toxic shock syndrome caused by group A streptococcus infection.

## Background

Streptococcal toxic shock syndrome (STSS) caused by *Streptococcus pyogenes*, a group A streptococcus (GAS), infection is an uncommon but serious illness that rapidly progresses to multiple organ failure, shock, and death. Therefore, it is important to promptly establish a diagnosis, provide hemodynamic support, and initiate appropriate antibiotics therapy. There are multiple prior reports of the use of extracorporeal membrane oxygenation (ECMO) in treatment of toxic shock in adults [1, 2]. Our case report is the first to demonstrate the successful treatment of cardiac arrest caused by STSS using ECMO and prompt initiation of antibiotic therapy. The rapid antigen GAS test may be an effective approach to promptly diagnose STSS caused by GAS infection.

## Case presentation

**Fig. 1:**
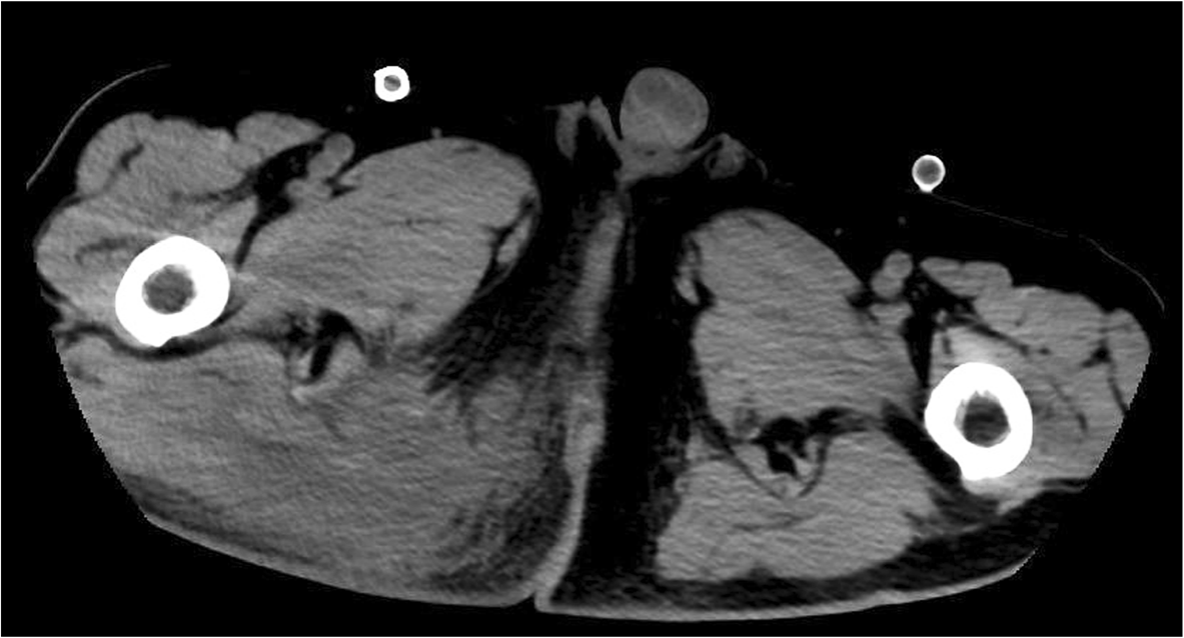
Computed tomography image of the pelvis showing a large low-density area in the right gluteus maximus muscle

A 70-year-old Asian man with ventricular fibrillation, who collapsed suddenly at a public bath, was brought to our hospital via ambulance. He had been prescribed allopurinol to treat gout for 15 years. No other relevant past history was found, including no history of diabetes or heart disease. He was an ex-tobacco smoker and drank one can (350 mL) of beer daily. ECMO was initiated 20 minutes after unsuccessful conventional resuscitation with five attempts of electrical cardioversion. On the sixth cardioversion attempt, sinus rhythm was achieved. His initial blood investigations showed the following: white blood cells, 70,510 cells/μL; hemoglobin, 14.3 mg/dL; platelets, 433,000 cells/μL; random blood sugar, 174 mg/dL; serum creatinine, 4.90 mg/dL; blood urea nitrogen, 82 mg/dL; serum glutamic-pyruvic transaminase, 76 IU/L; serum glutamic oxaloacetic transaminase, 58 IU/L; creatinine kinase 194 U/L; and serum C-reactive protein, 40.7 mg/dL (Table 1). Transthoracic echocardiography demonstrated diffuse hypokinesis, and anteroseptal and apical akinesis with impaired left ventricular function and an ejection fraction of 25%. Emergency coronary angiography revealed normal coronary arteries. On physical examination, a large abscess in his right gluteal region was detected; computed tomography showed a large low-density area in the right gluteus maximus muscle (Fig. 1).

**Table 1:** Laboratory data at hospital admission

WBCs	70,510	/μL	Total protein	7.9	g/dL
Neutrophils	85.6	%	Albumin	2.7	g/dL
Lymphocytes	9.1	%	Na	134	mEq/L
Monocytes	4.9	%	K	5.7	mEq/L
Basophils	0.1	%	Cl	94	mEq/L
Eosinophils	0.3	%	Glucose	174	mg/dL
Hemoglobin	14.3	g/dl	HbA1c	5.9	%
Platelets	43.3	× 10^4^/μL			
			Arterial blood gas^*^		
CRP	40.7	mg/dL	pH	6.939	
Total bilirubin	0.4	mg/dL	PaCO_2_	30.8	mmHg
AST	58	U/L	PaO_2_	66.4	mmHg
ALT	76	U/L	HCO_3_^−^	6.5	mmol/L
LDH	340	U/L	BE	−25.1	mmol/L
ALP	525	U/L			
γGTP	66	U/L			
CK	194	U/L			
Blood urea nitrogen	82	mg/dL			
Creatinine	4.9	mg/dL			

^*^Arterial blood gas was obtained at *10 L*/minute of *oxygen* via a face mask

*γ-GTP* gamma-glutamyl transpeptidase, *ALP* alkaline phosphatase, *ALT* alanine aminotransferase, *AST* aspartate aminotransferase, *BE* base excess, *CK* creatine kinase, *CRP* C-reactive protein, *HbA1c* glycated hemoglobin, *HCO*_*3*_^−^ hydrogen carbonate, *LDH* lactate dehydrogenase, *PaCO*_*2*_ partial pressure of carbon dioxide in arterial blood, *PaO*_*2*_ partial pressure of oxygen in arterial blood, *WBC* white blood cell count

**Fig. 2:**
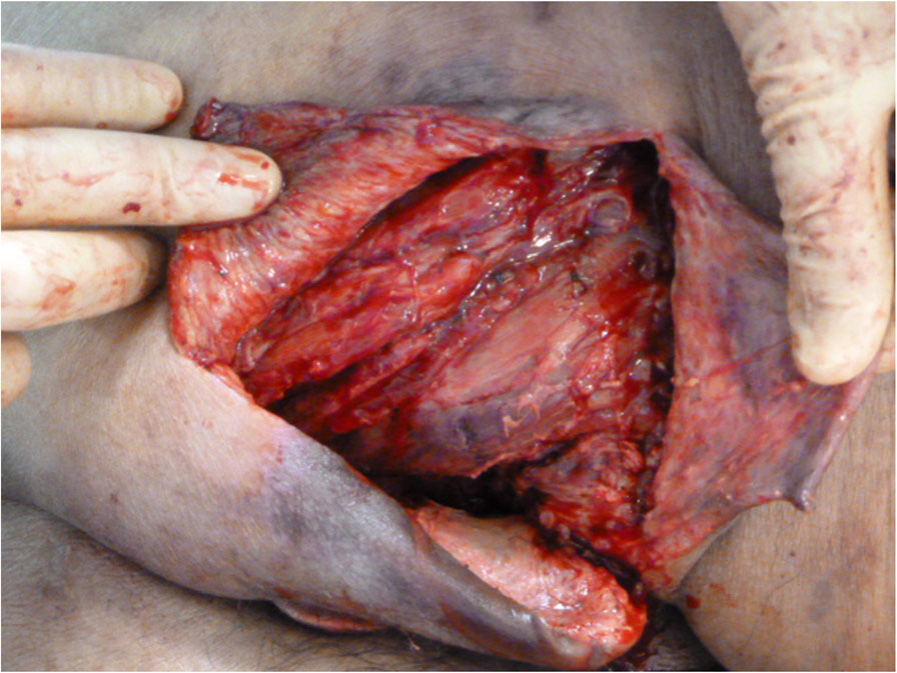
Surgical drainage of the abscess in the right gluteus maximus muscle

**Fig. 3:**
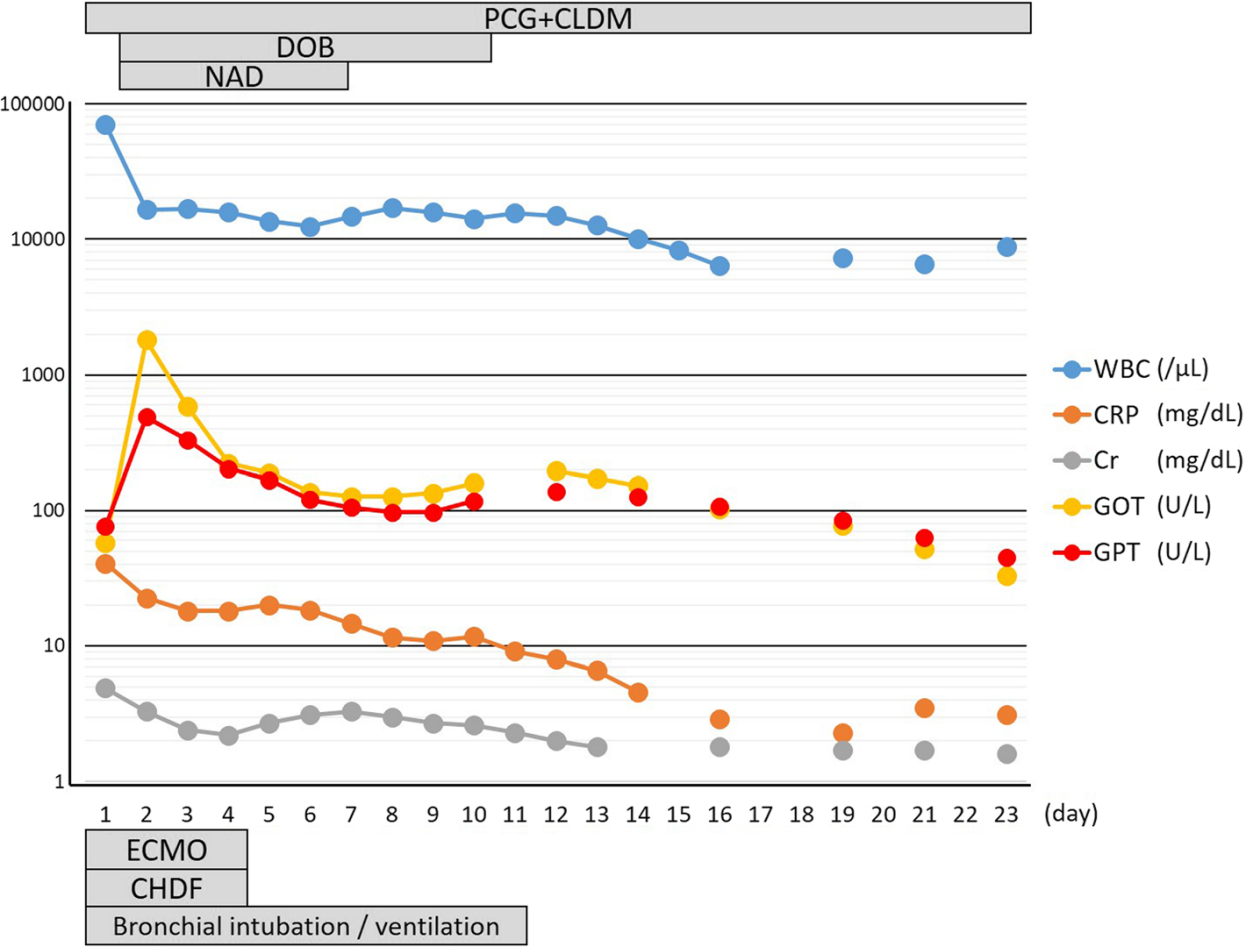
Clinical course with laboratory and treatment data. *CHDF* continuous hemodiafiltration, *CLDM* clindamycin, *Cr* creatinine, *CRP* C-reactive protein, *DOB* dobutamine, *ECMO* extracorporeal membrane oxygenation, *GOT* glutamic oxaloacetic transaminase, *GPT* glutamic-pyruvic transaminase, *NAD* noradrenaline, *PCG* penicillin G, *WBC* white blood cells

Thus, we diagnosed our patient as having septic shock due to a gluteal abscess and conducted surgical drainage (Fig. 2). Concurrently, a rapid antigen GAS test (Quick Chaser Dip Strep A®; Mizuho Medy Co., Japan) was performed using a sample obtained from the right gluteal abscess. Positive results were observed within a minute; therefore, antibiotic therapy comprising benzylpenicillin (1200 U/day) and clindamycin (1200 mg/day) was initiated immediately. Five days after admission, the culture of the purulent matter yielded *Streptococcus pyogenes*; thus, we diagnosed our patient as having STSS based on the criteria [3]. Subsequently, his general condition improved; he was successfully weaned from ECMO and continuous hemodiafiltration on day 4 and successfully weaned from ventilation on day 9. The intravenous administration of antibiotics was continued until day 37 since the initiation of therapy. He was discharged after receiving a skin graft on day 83 (Fig. 3). He had no clinical problem at 6 months after hospital discharge.

## Discussion

Here we reported a case of out-of-hospital cardiac arrest (OHCA) due to septic shock in a 70-year-old Asian man. To the best of our knowledge, this is the first case report to highlight the effectiveness of ECMO on cardiac arrest caused by rapidly progressing STSS. GAS is the most common and frequent causative pathogen of acute pharyngitis, accounting for 10–30% of cases in children and 5–10% of cases in adults [4]. However, streptococcal infections are also known to occasionally progress to STSS on rare occasions, in which patients show rapidly worsening clinical conditions and very poor prognoses. A retrospective study involving 3566 case-patients with severe *Streptococcus pyogenes* infection, in which 698 (20%) patients died within 30 days of culture-positive specimens being obtained, indicated that the survival probability was lowest among patients in whom STSS developed: 26% of patients with STSS died of septic shock within a day of specimen collection [5]. The utility of ECMO in adults with sepsis remains controversial. In the current case, the patient presented with cardiac arrest, which is resistant to conventional resuscitation; therefore, ECMO was introduced promptly. Although there is a paucity of data supporting the use of ECMO in this spectrum of pathologic conditions, several recent reports have suggested that ECMO can become a valuable therapeutic option for patients with refractory cardiovascular dysfunction, especially when introduced promptly after developing septic shock. A recent retrospective analysis of 151 adult patients with sepsis receiving ECMO claimed that worse outcomes were significantly associated with longer door-to-ECMO times, because delayed rescue leads to irreversible multiorgan failure [6]. Moreover, another study showed that the development of shock beyond 30.5 hours before ECMO initiation was associated with 0% survival. However, even after successful weaning from ECMO, the prognosis is reported to be poor. That study also reported that among 40.6% of the patients who were successfully weaned from ECMO, only half (21.9%) survived the refractory septic shock [7].

One of the possible explanations for such poor prognoses after successful weaning from ECMO is the delay in the identification of the pathogenic bacteria and the following treatment. A significant disadvantage of culturing a sample is the delay of 1–2 days to obtain results. This delay may cause initiation of inappropriate antibiotic therapy and could be fatal, especially in STSS owing to the rapid progression. Further, indiscriminate use of antibiotics may result in unnecessary adverse reactions, antibiotic resistance, and increased health care costs. Recently, high sensitivity-based and specificity-based optical immunoassay technologies for detecting GAS antigen have become available [8–10], in which the results can be obtained within 5–10 minutes. This could allow for prompt treatment of patients with STSS with the appropriate antibiotic and reduce the risk for overuse of antibiotic treatment in uncertain situations while culture results are pending. Early treatment may lead to more rapid improvement of the patient’s general condition, as seen in the current case.

## Conclusions

Our case report is the first to demonstrate the successful treatment of OHCA caused by STSS via ECMO and prompt initiation of antibiotic therapy. The findings of this case demonstrated that the rapid antigen GAS test, usually used for the diagnosis of GAS pharyngitis, was very effective to promptly diagnose STSS caused by GAS infection. Emergency physicians who encounter patients OHCA with septic shock should consider the possibility of GAS infection and therefore order a rapid antigen GAS test.

### Key summary points

- We reported a case of OHCA due to septic shock in a 70-year-old Asian man. To the best of our knowledge, this is the first case report to highlight the effectiveness of ECMO on cardiac arrest caused by rapidly progressing STSS.
- The utility of ECMO in adults with sepsis remains controversial. In the current case, the patient presented with cardiac arrest, which is resistant to conventional resuscitation; therefore, ECMO was introduced promptly.
- The rapid antigen GAS test, usually used for the diagnosis of GAS pharyngitis, was very effective to promptly diagnose necrotizing fasciitis and STSS caused by GAS infection.
